# TLR4/Inflammasomes Cross-Talk and Pyroptosis Contribute to N-Acetyl Cysteine and Chlorogenic Acid Protection against Cisplatin-Induced Nephrotoxicity

**DOI:** 10.3390/ph16030337

**Published:** 2023-02-22

**Authors:** Amira M. Badr, Layla A. Al-Kharashi, Hala Attia, Samiyah Alshehri, Hanaa N. Alajami, Rehab A. Ali, Yasmen F. Mahran

**Affiliations:** 1 Department of Pharmacology and Toxicology, College of Pharmacy, King Saud University, Riyadh 11211, Saudi Arabia; 2 Department of Pharmacology & Toxicology, Faculty of Pharmacy, Ain Shams University, Cairo 11566, Egypt; 3 Department of Biochemistry, Faculty of Pharmacy, Mansoura University, Mansoura 35516, Egypt

**Keywords:** cisplatin, nephrotoxicity, TLR4, inflammasomes, NLPR3, N-acetylcysteine, chlorogenic acid, gasdermin

## Abstract

Background: Cisplatin (Cp) is an antineoplastic agent with a dose-limiting nephrotoxicity. Cp-induced nephrotoxicity is characterized by the interplay of oxidative stress, inflammation, and apoptosis. Toll-4 receptors (TLR4) and NLPR3 inflammasome are pattern-recognition receptors responsible for activating inflammatory responses and are assigned to play a significant role with gasdermin (GSDMD) in acute kidney injuries. N-acetylcysteine (NAC) and chlorogenic acid (CGA) have documented nephroprotective effects by suppressing oxidative and inflammatory pathways. Therefore, the current study aimed to investigate the contribution of the upregulation of TLR4/inflammasomes/gasdermin signaling to Cp-induced nephrotoxicity and their modulation by NAC or CGA. Methods: A single injection of Cp (7 mg/kg, i.p.) was given to Wistar rats. Rats received either NAC (250 mg/kg, p.o.) and/or CGA (20 mg/kg, p.o.) one week before and after the Cp injection. Results: Cp-induced acute nephrotoxicity was evident by the increased blood urea nitrogen and serum creatinine and histopathological insults. Additionally, nephrotoxicity was associated with increased lipid peroxidation, reduced antioxidants, and elevated levels of inflammatory markers (NF-κB and TNF-α) in the kidney tissues. Moreover, Cp upregulated both TLR4/NLPR3/interleukin-1beta (IL-1β) and caspase-1/GSDMD-signaling pathways, accompanied by an increased Bax/BCL-2 ratio, indicating an inflammatory-mediated apoptosis. Both NAC and/or CGA significantly corrected these changes. Conclusions: This study emphasizes that inhibition of TLR4/NLPR3/IL-1β/GSDMD might be a novel mechanism of the nephroprotective effects of NAC or CGA against Cp-induced nephrotoxicity in rats.

## 1. Introduction

Cisplatin (cis-diamminedichloroplatinum II, Cp) is one of the most effective antineoplastic agents, used to treat a variety of malignant tumors [1]. It has been known that Cp exerts its cytotoxic effect through alkylating the DNA double helix, resulting in intra-strand and inter-strand adducts, which explains why it inhibits cell division and has the greatest efficacy in rapidly reproducing cells [2]. Despite its potential effectiveness in prolonging the patient survival rate, its dose-dependent nephrotoxicity is still not completely resolved [3,4]. Cp nephrotoxicity is encountered in 30–45% of patients receiving Cp; accordingly, the discontinuation of therapy and limitation of the overall clinical outcome will be the final result [5,6]. Therefore, there is an urgent need for a comprehensive understanding of the Cp-induced nephrotoxic-signaling pathways and optimal nephroprotective treatment to increase the clinical applicability of Cp.

Cp-induced nephrotoxicity is a complex process involving an interplay between oxidative stress, inflammation, and apoptosis [1,6]. In this context, it has been reported that acute kidney injury (AKI) and hyperuricemia are among the common clinical manifestations of Cp nephrotoxicity [7].

Studies have gradually uncovered the complex inflammatory pathways that are involved in Cp-induced AKI [8,9], e.g., Toll-4 receptors (TLR4) and inflammasomes. TLR4 and NLR family pyrin domain-containing 3 (NLRP3) inflammasomes are pattern recognition receptors responsible for activating inflammatory responses. They were recently assigned to play a plausible role in kidney injuries [9,10,11,12,13]. The expression of NLPR3, as well as pro-interleukin (IL)-1beta (pro-IL-1β), is triggered by nuclear factor-kB (NF-kB). NF-kB is activated by various stimuli, such as lipopolysaccharides binding to TLR4 and reactive oxygen species (ROS). NLRP3-mediated caspase-1 activation induces the cleavage of pro-IL-1β and pro-IL-18, which are recently known to be implicated in Cp-induced nephrotoxicity [13,14]. Accordingly, the inhibition of NLRP3 was found to ameliorate Cp-induced tissue injuries not only in the kidney, but also in the liver [13,15].

In addition to activating IL-β, caspase-1 can activate and cleave gasdermin D (GSDMD). GSDMD has the ability to form pores in the cell membranes, with the resultant leakage of cytoplasmic contents and inflammatory mediators, including IL-1β and IL-18, inducing a type of inflammatory cell death known as pyroptosis; the Greek root “pyro” means fever, and it is a sign of inflammation. Pyroptosis is a type of programmed cell death that is activated by inflammatory caspases, mainly caspase-1 [16,17]. Pyroptosis differs from apoptosis in many aspects, including function and cell morphology. However, the main hallmark is that in pyroptosis, DNA fragmentation occurs, while the nucleus remains intact [16,18]. Pyroptosis was found to be implicated in the development of kidney diseases, including ischemia-reperfusion injury [19,20], diabetic nephropathy [21], fibrosis [22], and lupus nephritis [18]. Accordingly, the activation of caspase-1/GSDMD pathway was documented to play a role in pyroptotic inflammatory responses of Cp-induced AKI [17]. Additionally, one study recently reported that Cp might have induced pyroptosis in breast cancer through an NLRP3/caspase-1/GSDMD pathway [23].

In addition to pyroptosis, apoptotic cell death also plays a role in the development of renal diseases [24]. The rate of apoptosis of kidney tubular epithelial cells is the key pathophysiological alteration occurring during ischemia/reperfusion, and it determines the level of kidney damage [25]. Moreover, diabetic kidney disease is also associated with tubular atrophy and epithelial cell apoptosis [24]. Cisplatin nephrotoxicity is also associated with the induction of apoptosis, even at low concentrations. This is mostly associated with the production of ROS, which damages mitochondrial membrane lipids and culminates in increased caspase-3 activity. Additionally, nephroprotection mediated by various pharmacological modalities is associated with a reduction in caspase-3 activity [26,27,28,29,30], further confirming the role of caspase-3 in Cp-induced nephrotoxicity [26,28,29,30].

The use of antioxidant and anti-inflammatory drugs to help minimize Cp-induced nephrotoxicity has gained considerable interest [31]. N-acetylcysteine (NAC), the acetylated variant of the amino acid L-cysteine, has a well-documented antioxidant and anti-inflammatory activity and is widely used as an antidote for acetaminophen toxicity [32,33]. Chlorogenic acid (CGA) is a phenolic compound widely found in fruits, vegetables, coffee, and tea, and it also has well-characterized antioxidant and anti-inflammatory properties [34,35]. Indeed, the antioxidant and anti-inflammatory properties of NAC [36,37] and CGA [38,39] have been demonstrated to promote nephroprotection. Few studies have recently linked the inflammasome pathway to their hepatoprotective effect [40,41]. However, no data is available about the contribution of TLR4/inflammasomes/pyroptosis signaling inhibition to NAC and CGA nephroprotective activity. Therefore, the present study was conducted to answer the upcoming questions: (1) Does TLR4 crosstalk with NLPR3/IL-1β signaling in Cp-induced AKI, and if so; (2) Does the inhibition of TLR4 and NLRP3/IL-1β signaling play a role in NAC- and CGA-mediated nephroprotection against Cp-induced nephrotoxicity, and how is this nephroprotection effect associated with caspase 1/GSDMD-induced pyroptosis?

## 2. Results

### 2.1. NAC and/or CGA Ameliorated Cp-Induced Nephrotoxicity

To confirm the nephroprotective effect of NAC and/or CGA on the acute nephrotoxicity induced by a single dose of Cp, biochemical renal function indices were measured, and a histopathological examination was conducted. Table 1 shows that Cp increased the kidney index by 42% compared to the control group. In addition, Cp caused a significant rise in both BUN and serum creatinine to about 778%, *p* = 0.0001 and 903%, *p* = 0.0003, respectively, compared to the control values. However, the administration of NAC or CGA significantly decreased BUN (by 74% and 65%, respectively) and serum creatinine (by 55% and 47%, respectively) compared to the Cp group. In addition, the combined group showed superior correction of both BUN and serum creatinine over NAC or CGA (by 82% and 60%, respectively). Nevertheless, no significant changes have been found in NAC, CGA, or the combined groups in values of kidney indexes when compared to the Cp group.

**Figure 1:**
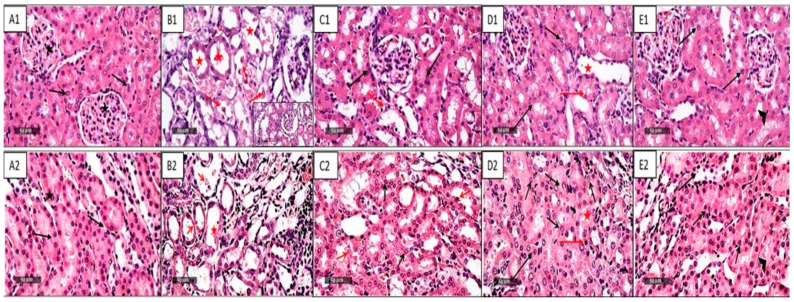
Histological Photomicrographs of the Kidney Specimens from Different Treatment Groups Stained with H and E. (**A1**,**A2**): Control group shows normal histological structure of the kidney glomeruli and renal tubules with the outer medullary region (**A2**); (**B1**,**B2**): Cisplatin-injected group shows severe tubular degenerative changes with cystic dilations and necrotic tubular segments of proximal and distal tubules (red star) and necrotic epithelial cells (red arrow) with mild interstitial inflammatory cells infiltrates; (**C1**,**C2**): NAC group (250 mg/kg/day p.o.) shows almost intact renal parenchyma and tubular epithelium (black arrow) with few focal records of degenerated tubular cells and pyknotic nuclei (red arrow); (**D1**,**D2**): CGA group (20 mg/kg/day p.o.) appears almost intact renal parenchyma and tubular epithelium (black arrow) with minimal records of dilated (red star) or degenerated tubular segments (red arrow), and minimal inflammatory cells infiltrate; (**E1**,**E2**): Combined group (NAC and CGA) shows apparent intact renal parenchyma and tubular epithelium (black arrow) with minimal dilatation of a few tubular segments, as well as occasional periglomerular inflammatory cells infiltrates (arrowhead), 400×. NAC: N-acetylcysteine; CGA: Chlorogenic acid.

Besides renal biochemical functions, histopathological alterations in kidney specimens were assessed using H &E staining, as shown in Figure 1. No histopathological alterations were found in the control group; it shows regular histological features of renal parenchyma with apparent intact renal corpuscles (star) and renal tubular segments with almost intact tubular epithelium (arrow), as well as intact vasculatures (Figure 1(A1,A2)).

On the other hand, the injection of Cp shows diffuse records of severe tubular degenerative changes at corticomedullary junctions with abundant figures of cystic dilations and necrotic tubular segments in both proximal and distal tubules (red star), with moderate forms of intraluminal desquamated necrotic epithelial cells (red arrow) with mild interstitial inflammatory cells infiltrates (Figure 1(B1,B2)). Meanwhile, the administration of NAC shows almost intact morphological features of renal parenchyma and tubular epithelium (black arrow) with few focal records of degenerated tubular cells with pyknotic nuclei (red arrow) (Figure 1(C1,C2)). In contrast, the CGA group demonstrates almost intact morphological features of renal parenchyma and tubular epithelium (black arrow) with minimal records of dilated (red star) or degenerated tubular segments (red arrow), as well as minimal inflammatory cells infiltrate (Figure 1(D1,D2)). Moreover, a combined group of both NAC and CGA shows apparent intact renal parenchyma and tubular epithelium (black arrow) with minimal dilatation of a few tubular segments, as well as occasional periglomerular inflammatory cells infiltrates (arrowhead) (Figure 1(E1,E2)). The scoring of renal tissue damage was represented in Table 2. Collectively, these results indicate that NAC and CGA alleviates Cp-induced nephrotoxicity.

### 2.2. NAC and/or CGA Reduced Cp-Induced Oxidative Stress

As shown in Table 1, seven days post-Cp injection, renal tissues showed massive oxidative stress confirmed by the upregulation of lipid peroxidation (reaching about 203%, *p* = 0.0001) and GPx activity (approximately 1480%, *p* = 0.0001) as well as the significant reduction of antioxidants and catalase activity (approximately 57%, *p* = 0.0069, and approximately 19%, *p* = 0.0001, respectively) compared with renal tissues of the control group. Nonetheless, both NAC and CGA counteracted the oxidative stress marker changes. NAC and CGA nearly normalized the levels of malondialdehyde and antioxidants and improved the cisplatin-induced changes in GPx levels. In addition, NAC and CGA increased the catalase activity to about 248% and 168% of the Cp group, respectively. The combined group showed approximately the same results as either the NAC- or the CGA-treated group.

### 2.3. Effect of NAC and/or CGA on Cp-Induced Inflammatory Signalling Responses

**Figure 2:**
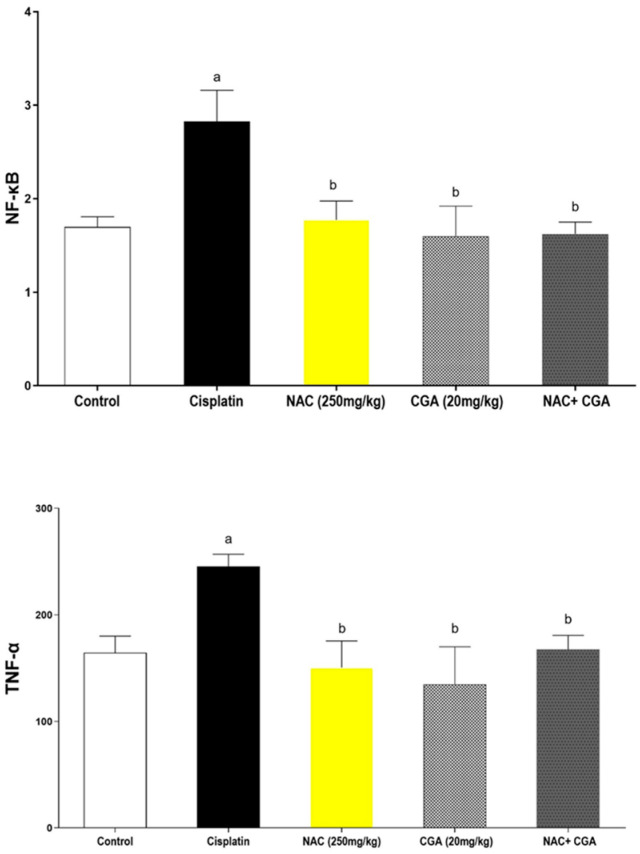
Effects of NAC and/or CGA on Cisplatin-Mediated Changes in Renal Expressions of NF-kB and TNF-α. Control: normal rats; Cisplatin: Cisplatin (7 mg/kg, IP) injected rats; NAC (250 mg/kg): Cisplatin-injected rats treated with NAC (250 mg/kg/day p.o.) one week before and after cisplatin injection; CGA (20 mg/kg): Cisplatin-injected rats treated with CGA (20 mg/kg/day p.o.) one week before and after cisplatin injection; NAC + CGA: Cisplatin-injected rats treated with NAC (250 mg/kg/day p.o.) and CGA (20 mg/kg/day p.o.) one week before and after cisplatin injection. Values are mean ± SEM (n = 5). ^a^: compared to the control group; ^b^ compared to the cisplatin group. For NF-kB (F = 5.58, df = 4, *p* = 0.0067) and for TNF-α (F = 5.56, df = 4, *p* = 0.0025). *p* values were evaluated using one-way ANOVA followed by Tukey–Kramer as a post-hoc test. NF-kB: Nuclear factor-κB; TNF-α: Tumor necrosis factor alpha; NAC: N-acetylcysteine; CGA: Chlorogenic acid.

The expression of the common inflammatory markers was assessed in kidney homogenates of different groups, and the results were represented in Figure 2. The renal expressions of NF-κB and TNF-α demonstrated a distinct upregulation in the Cp-treated group compared to the control group (166%, *p* = 0.0067 & 148%, *p* = 0.0025, respectively). However, the administration of NAC and/or CGA significantly reduced inflammatory responses of Cp in terms of NF-κB (37% reduction for NAC group, 43% reduction for CGA group, and 42% reduction for the combined group). At the same time, both NAC and/or CGA corrected this increment of TNF-α by approximately 38.7% for the NAC-treated group, 45% for the CGA-treated group, and 32% for the combination group.

### 2.4. NAC and/or CGA Inhibited the TLR4/NLPR3/IL-1β and Caspase-1/GSDMD Signaling in Cp-Induced Nephrotoxicity

**Figure 3:**
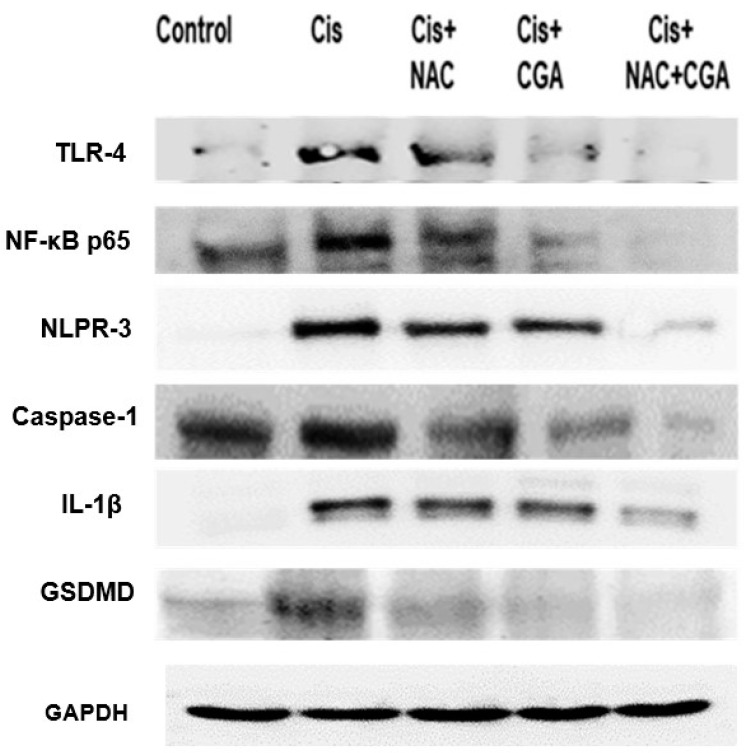
Effect of NAC and/or CGA on the TLR4/NLPR3/IL-1β and caspase-1/GSDMD Signaling Pathway in Kidney Tissue of Rats. Representatives immunoblot of protein level of TLR4, NF-κB, NLPR3, caspase-1, IL-1β, and GSDMD in kidney tissues. Sample proteins were immunoblotted with the antibodies’ “upper panels” and after stripping with GAPDH antibody as a loading control “lower panel.” Control: normal rats; Cis: Cisplatin (7 mg/kg, IP) injected rats; Cis + NAC: Cisplatin injected rats treated with NAC (250 mg/kg/day p.o.) one week before and after cisplatin injection; Cis + CGA: Cisplatin-injected rats treated with CGA (20 mg/kg/day p.o.) one week before and after cisplatin injection; Cis + NAC + CGA: Cisplatin-injected rats treated with NAC (250 mg/kg/day p.o.) and CGA (20 mg/kg/day p.o.) one week before and after cisplatin injection. NAC: N-acetylcysteine; CGA: Chlorogenic acid; TLR4: Toll-4 receptors; NF-κB: Nuclear factor kappa B; NLPR3: Inflammasomes; IL-1β: Interleukin-1 beta; GSDMD: Gasdermin D.

**Figure 4:**
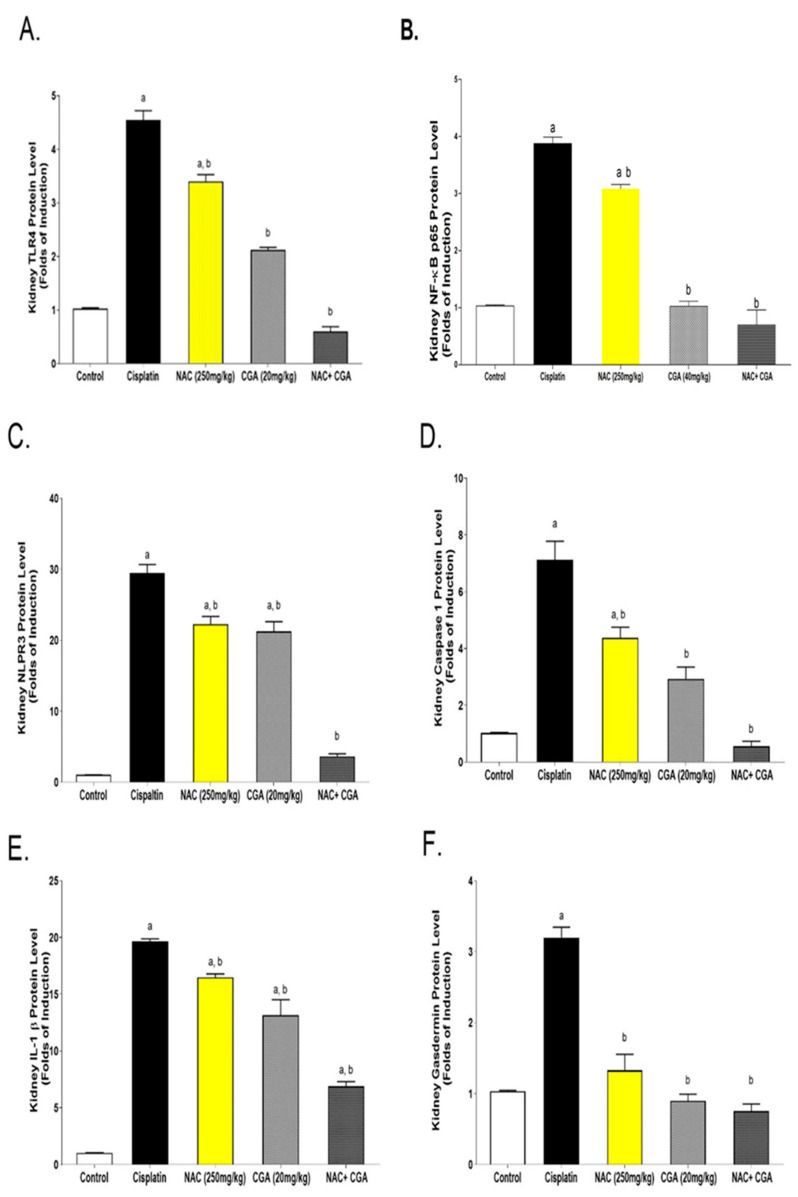
Quantitative Results of Immunoblots of TLR4/NF-kB/NLPR3/IL-1β and Caspase1/GSDMD Signaling Pathway in Kidney Tissue of Rats. Densities were quantified using analysis software. (**A**–**F**) Quantitative results of the immunoblot of TLR4, NF-κB, NLPR3, caspase-1, IL-1β, and GSDMD. Protein levels are expressed as the ratio of protein/GAPDH. The relative quantities were normalized to the control and expressed as a fold of induction. Data are presented as the mean ± SEM (n = 3). ^a^ *p* ≤ 0.05 compared to the control group; ^b^ *p ≤* 0.05 compared to the cisplatin group. *p* ≤ 0.05 using ANOVA followed by Tukey-Kramer as post-hoc test. Control: normal rats; Cis: Cisplatin-injected (7 mg/kg, i.p.) rats; Cis + NAC: Cisplatin-injected rats treated with NAC (250 mg/kg/day, p.o.) one week before and after cisplatin injection; Cis + CGA: Cisplatin-injected rats treated with CGA (20 mg/kg/day, p.o.) one week before and after cisplatin injection; Cis + NAC + CGA: Cisplatin-injected rats treated with NAC (250 mg/kg/day, p.o.) and CGA (20 mg/kg/day, p.o.) one week before and after cisplatin injection. For TLR4 (F = 238.8, df = 4, *p* < 0.0001), for NF-kB (F = 226, df = 4, *p* < 0.0001), for NLPR3 (F = 144, df = 4, *p* < 0.0001), for caspase-1 (F = 41, df = 4, *p* < 0.0001), for IL-1β (F = 125.3, df = 4, *p* < 0.0001), and for GSDMD (F = 50, df = 4, *p* < 0.0001). *p* values were evaluated using one-way ANOVA followed by Tukey–Kramer as a post-hoc test. NAC: N-acetylcysteine; CGA: Chlorogenic acid; TLR4: Toll-4 receptors; NF-κB: Nuclear factor kappa B; NLPR3: Inflammasomes; IL-1β: Interleukin-1 beta; GSDMD: Gasdermin D.

To further explore the molecular mechanisms underlying Cp-induced acute nephrotoxicity, the fundamental role of the TLR4/NLPR3/IL-1β pathway, along with the GSDMD and caspase-1, was investigated using western blot analysis of the protein expression of TLR4, NF-κB, NLPR3, caspase-1, and IL-1β, as shown in Figure 3 and Figure 4. The resulting immunoblotting analysis in the Cp-treated group showed sharp upregulation in protein expression levels (4.4 folds for TLR4, 3.7 folds for NF-κB, 28.6 folds for NLPR3, seven folds for caspase-1, and 19 folds for IL-1β) when compared with the control group (Figure 3 and Figure 4A–E). On the other hand, the treatment of Cp-injected rats with 250 mg/kg NAC or 20 mg/kg CGA one week before and after Cp injection significantly reduced the upregulation of these protein expressions. However, rats treated with both NAC and CGA showed superior downregulation of those proteins (about 80%-reduction for TLR4, 80%fold reduction for NF-κB, 80% reduction for NLPR3, 68% reduction in caspase-1, and 55% reduction for IL-1β) and protein expression levels compared to the Cp-group (Figure 3 and Figure 4A–E).

Concomitantly, Figure 3 and Figure 4F show western blot analysis of GSDMD. The intense induction of both proteins’ expressions occurred following Cp injection (3-fold increment for GSDMD) compared to the control values. However, NAC and/or CGA administration corrected this increment and nearly normalized the proteins’ expression levels, as shown in Figure 3 and Figure 4F.

### 2.5. Inhibition of Apoptotic Markers by NAC’s and/or CGA’s

**Figure 5:**
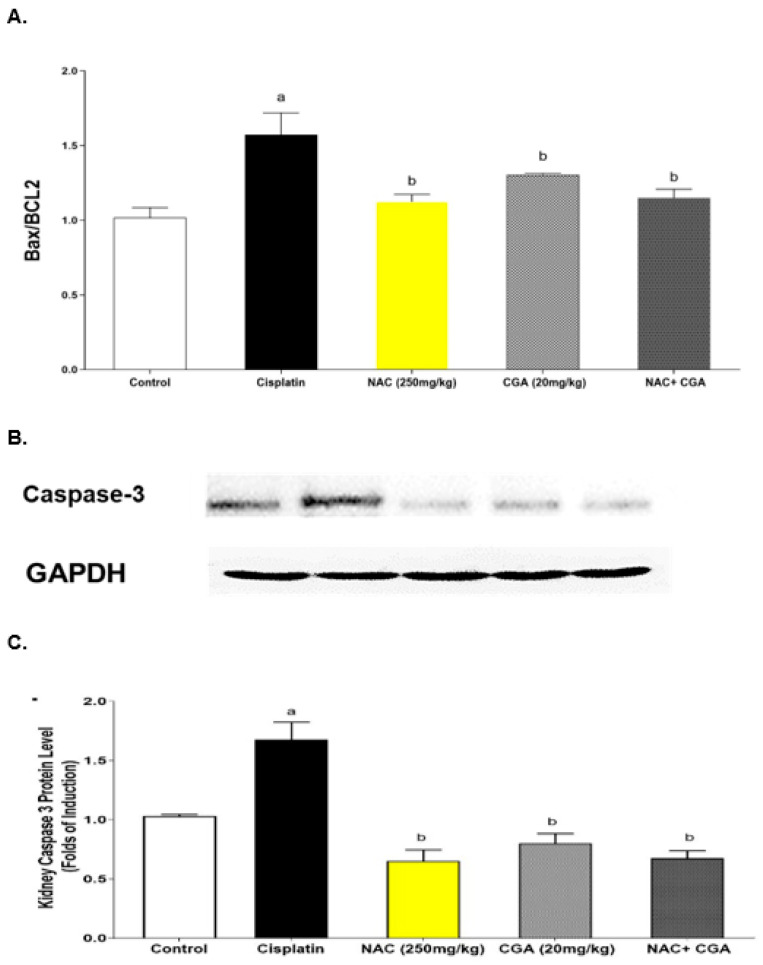
Effect of NAC and/or CGA on Cisplatin-Induced upregulation in Apoptotic Markers: (**A**): Effect of NAC and/or CGA on cisplatin-induced changes in Bax/Bcl-2 ratio, values are mean ± SEM, n = 5. (**B**): Effect of NAC and/or CGA on cisplatin-induced upregulation of caspase-3 using immunoblot. (**C**): Quantitative results of immunoblots of caspase-3, values are mean ± SEM, n = 3. Protein levels are expressed as the ratio of protein/GAPDH. The relative quantities were normalized to the control and expressed as a fold of induction (F = 19.84, df = 4, *p* < 0.0001). Control: normal rats; Cisplatin: Cisplatin-injected rats (7 mg/kg, i.p.); NAC (250 mg/kg): Cisplatin-injected rats treated with NAC (250 mg/kg/day p.o.) one week before and after cisplatin injection; CGA (20 mg/kg): Cisplatin-injected rats treated with CGA (20 mg/kg/day, p.o.) one week before and after cisplatin injection; NAC + CGA: Cisplatin-injected rats treated with NAC (250 mg/kg/day, p.o.) and CGA (20 mg/kg/day, p.o.) one week before and after cisplatin injection. ^a^ *p* < 0.05 compared to the control group; ^b^ *p* < 0.05 compared to the cisplatin group. *p* value was evaluated using one-way ANOVA followed by Tukey–Kramer as a post-hoc test. Bax/Bcl2: Bax/Bcl2 ratio; NAC: N-acetylcysteine; CGA: Chlorogenic acid.

The involvement of apoptotic pathways in Cp-mediated nephrotoxicity has been documented. Therefore, the Bax/BCL2 ratio was assessed to explore the renoprotective mechanisms of NAC and/or CGA. At the end of the experiment, the Cp-injected rats demonstrated intense upregulation of the Bax/BCL2 ratio (to about 1.57-fold, *p* = 0.0024) compared to the control rats (Figure 5A). On the other hand, NAC and/or CGA administration one week before and after the Cp injection significantly downregulated the Bax/BCL2 ratio to about 1.12-fold for the NAC group, 1.3-fold for the CGA group, and 1.15-fold for the combined treatment group compared to the Cp-treated rats (Figure 5A). Caspase-3 renal expression was increased by Cp by about 1.6 folds compared to the control group (Figure 5B,C). However, NAC and/or CGA administration corrected this increment and nearly normalized the proteins’ expression levels, as shown in Figure 5B,C.

## 3. Discussion

Cp has been known for its crucial role in prolonging patients’ survival rates, particularly those with solid tumors. However, the discontinuation of therapy because of its massive nephrotoxicity has limited its clinical outcome for decades. Although scientists have been trying to alleviate this acute nephrotoxicity through enormous measures, no ideal nephroprotective agent has emerged, and the molecular mechanisms have not been fully elucidated [1,2,4,42]. Indeed, Ozkok and Edelstein [42] suggested that the deep studying of the pathogenesis of Cp-mediated AKI is vital to prevent such AKI and improve survival in cancer patients receiving Cp. Therefore, the present study was the first to elaborate on the potential role of TLR4/inflammasome signaling in the nephroprotection provided by NAC and CGA against Cp-induced acute nephrotoxicity in rats. The possible nephroprotective mechanisms of NAC and CGA were investigated, including their effects on oxidative and inflammatory status, as well as pyroptosis and apoptosis.

In the present study, a single intraperitoneal injection of Cp (7 mg/kg) resulted in increased BUN, serum creatinine, and kidney indices. Cp also induced severe tubular degeneration, cystic dilations, and necrotic tubular segments, which were consistent with increased renal function indices and severe necrosis and inflammation of glomerular and tubular cells, as previously reported [43]. These findings confirm massive morphological damage and functional disablement in the kidney that leads to the accumulation of BUN and serum creatinine due to the inability of the kidney to clear nitrogenous substances [44]. Our findings confirmed those of previous studies [45,46]. The administration of NAC and CGA markedly hampered these nephrotoxic damages supporting their promising nephroprotective role, and our results were consistent with previous studies [37,47].

In addition to nephrotoxicity markers, the molecular mechanisms underlying NAC and CGA nephroprotection were fully investigated. There is undeniable evidence that oxidative stress, inflammation, and apoptosis are among the confirmed pathophysiological responses of Cp-induced nephrotoxicity. Cp induces extensive mitochondrial-ROS response in renal tissues and forms protein- and thiol-addition products, resulting in the depletion of antioxidant resistance [48,49]. The generated oxidants/antioxidants imbalance promotes cellular damage [49], oxidative stress-mediated apoptosis and inflammation [50,51]. In this study, the Cp-injected rats demonstrated deliberate oxidative stress in the kidneys indicated by a significant increase in lipid peroxidation along with the depletion of total antioxidants levels and reduced catalase activity. These findings were consistent with those reported by previous studies [27,51,52,53]. ROS can activate both inflammatory and apoptotic pathways. Studies have proven the contribution of the inflammatory signaling responses in the pathogenesis of Cp-mediated renal tubular injuries. Our current study confirmed the inflammatory response induced by Cp injection, evidenced by the upregulation of tissue expression of NF-κB, TNF-α, and IL-1β [54,55].

Moreover, our results proved that Cp-induced massive apoptosis in renal tissues, as indicated by a significant escalation in the expression of the executive caspase, caspase-3, and the ratio of the proapoptotic protein (Bax) to the antiapoptotic one (BCL2). Cp-induced apoptosis most probably linked to ROS-induced mitochondrial injury, which results in the activation of the intrinsic apoptotic pathway [51,56]. Cp-induced apoptosis may also be a consequence of increased TNF-α, which can trigger the extrinsic apoptotic pathway via activating the Death Receptors. Our findings were consistent with what was reported previously [55,57,58,59]. Accordingly, TNF-α inhibitors showed a promising role in alleviating Cp-induced renal damage [57,59].

The current results showed that both NAC and CGA substantially hampered the renal tubular inflammation and apoptosis indicated by a significant reduction in NF-κB, TNF-α level, caspase-3 expression, and Bax/BCL2 ratio compared to the Cp group. These results were in accordance with recent studies [60,61]. We suggest that NAC and CGA might mitigate the Cp-induced apoptotic response by rebalancing renal tubular cells’ oxidative stress status.

The current study aimed to further elucidate the inflammatory and apoptotic molecular mechanisms involved in Cp-induced nephrotoxicity. Both TLR4 and inflammasomes were recently reported to play a role in Cp-induced nephrotoxicity [62,63]. However, their cross-talk role in the NAC- and CGA-nephroprotective activity was not previously studied. TLR4 and inflammasomes were cross-talked in other disease models, e.g., Alzheimer’s disease [64]. Therefore, we hypothesized in this study that both TLR4 and inflammasomes are upregulated and cross-talked in Cp-induced nephrotoxicity. They play a crucial role in NAC and CGA nephroprotection.

Both TLR4 and inflammasomes are types of pattern recognition receptors (PRR) that are capable of recognizing molecules frequently found in pathogens (including bacteria and viruses) or molecules released by damaged cells; they are known as pathogen-associated molecular patterns (Pamps) and endogenous-associated molecular patterns (Damps). Once activated, they initiate pro-inflammatory responses required to eliminate infectious agents or induce cell death [64]. The inflamed necrotic tubules release damage-associated molecular pattern molecules [65], which bind to TLR4 and induce different inflammatory responses, through the NF-κB pathway [66]. This results in a marked elevation in the levels of TNF-α and pro-IL-1β. Eventually, the pro-IL-1β is activated by an NLRP3, a cytosolic PRR. Once the NLRP3 gets activated, it oligomerizes and subsequently binds to the pro-caspase-1 and the adapter protein (ASC), forming a large inflammasome complex. This, in turn, results in the activation of pro-caspase-1, as well as the cleavage of the pro-IL-1β into the active IL-1β [11,67].

Swanson et al. [67] have suggested that NLRP3 activation and subsequent IL-1β production require two fundamental regulatory steps: priming and activation. In the priming step, NF-κB triggers the upregulation of the expression of the inflammasome components (NLRP3, caspase-1, and pro-IL-1β) to the level required for the activation step. In this context, NF-κB activation can be induced through a TLR4 dependent pathway [64,67]. The activation of inflammasomes can occur in several ways, including increased Pamps or Damps; Damps include ROS, mitochondrial damage, calcium influx, or potassium efflux. IL-1β is secreted outside the cell, where it can bind to IL-1β receptors to induce further NF-κB expression and other inflammatory responses inside the cell [68].

**Figure 6:**
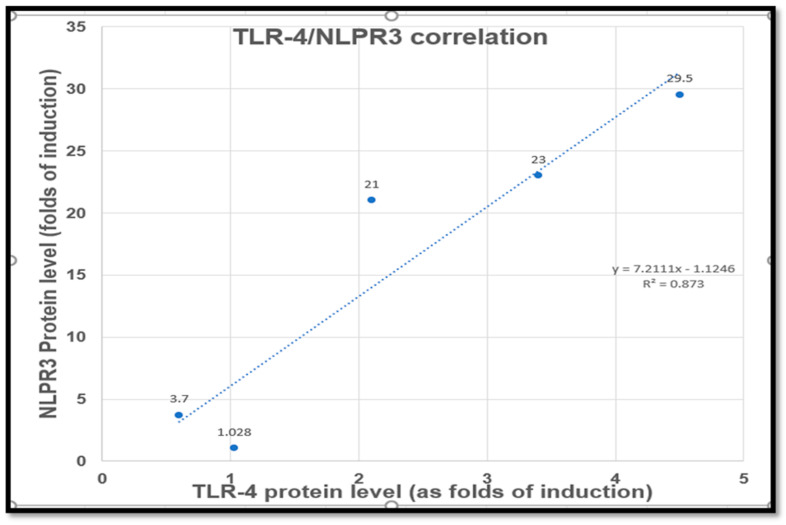
Correlation of TLR4 and NLPR3 protein expression. TLR4: Toll-4 receptors, NLPR3: Inflammasomes.

Aiming to answer the central questions of this current study, the TLR4/NLRP3 signaling pathway was investigated using western blot analysis. This study confirmed the involvement of the TLR4/NLRP3/IL-1β signaling in Cp-induced renal tubular damage. This is evidenced by the upregulation of the upstream proteins’ renal expression involved in the inflammasomes pathway’s priming step, such as NF-κB, TLR4, and NLRP3. In addition, intense increments in IL-1β and caspase-1 renal expressions were detected following Cp injection, and these findings were consistent with previous studies [69,70]. Interestingly, NAC and CGA administration reversed the upregulation of the inflammasome signaling responses and were accompanied by the correction of renal function, as well as oxidative and inflammatory indices. Moreover, a simple correlation curve comparing TLR4 and NLPR3 expression in different groups demonstrated a probable strong correlation (R^2^ = 0.87) between TLR4 and NLPR3 expression, Figure 6. Our current study suggested that the suppression of the TLR4/NLRP3-signaling pathway is one of the mechanisms incorporated in the nephroprotection of NAC and CGA against Cp-induced renal insults.

Furthermore, few studies investigated the role of the caspase-1/GSDMD pathway in Cp-induced pyroptosis and inflammation-mediated programmed cell death [17]. Recently, GSDMD was identified as a critical mediator of pyroptosis. Active caspase-1 cleaves GSDMD within a linker between its N-terminal and C-terminal domains. After cleavage, the N-terminal field forms pores in the cell membrane to cause pyroptosis. In the current study, the protein expression of GSDMD in kidney tissues was examined in the Cp-injected rats, and the results proved that the upregulation of both caspase-1 was associated with the upregulation of GSDMD renal expression. Cp-induced pyroptosis was found to play a role in both Cp-cytotoxic effects [71,72], as well as its associated nephrotoxicity [13,17], cardiotoxicity [73], and cochlear toxicity [74]. In harmony, in the current study, Cp induced a significant increase in GSDMD compared to the control group. The results are in agreement with that reported before [13,28]. Treatment with NAC and/or CGA significantly inhibited GSDMD. For the first time, this study suggests that the crosstalk of TLR4 and NLRP3 might be implicated in Cp-induced nephrotoxicity, with the consequent activation of caspase-1/GSDMD. Additionally, NAC and CGA could protect kidney tissues from Cp-induced inflammatory mediated cell-death through the suppression of TLR4/NLRP3/caspase-1/GSDMD signaling and thus inhibiting pyroptosis, as well as apoptosis.

The limitation and future recommendation based on this study is the need for studying the effect of NAC and CGA on Cp-induced cytotoxicity. The data is limited regarding NAC [75,76], and CGA [77,78], and they are not conclusive.

## 4. Material and Methods

### 4.1. Materials

#### 4.1.1. Drugs and Chemicals

Cisplatin [Onco-Tain^®^] was purchased from Hospira, UK, LTD. Chlorogenic acid hemihydrate (ab120973) was obtained from Abcam Co. (Cambridge, MA, USA). N-acetylcysteine was purchased from Sigma (St. Louis, MO, USA). Tris buffer, non-fat dry milk, bovine serum albumin, and RIPA buffer were obtained from Sigma (St. Louis, MO, USA). Other chemicals were of the highest grade commercially available.

#### 4.1.2. Animals

In this study, we followed the ethical guidelines of the Faculty of Pharmacy, King Saud University, Saudi Arabia. Ethical approval was issued form King Saud Scientific Research Ethics Committee (IRB number: KSU-SE-20-52). Male Wistar rats (150–200 g), 10 weeks old in average, were obtained from the animal house of the Faculty of Pharmacy, King Saud University, Riyadh, Saudi Arabia, and were acclimated for one week before experimentation in standard conditions: air-conditioned atmosphere, 25 °C, and 12-h light and dark alternative cycles. In addition, rats were provided with a standard diet and water ad libitum. According to the legal guidelines, standard diet pellets contained not less than 20% protein, 5% fiber, 3.5% fat, and 6.5% ash, as well as a vitamin mixture.

### 4.2. Methods

#### 4.2.1. Experiment Design and Sample Collection

Forty rats were randomly assigned into five groups, with eight animals per group (n = 8) and treated for 14 days as follows: **Group 1 (control group)**—rats were given only vehicle (distilled water) daily by oral gavage for 14 days. **Group 2 (Cp group)**—rats were given vehicle daily by oral gavage for 14 days and injected with a single injection of Cp (7 mg/kg, i.p.,) on Day 7 to induce the nephrotoxicity. **Group 3 (NAC group)**—rats received a daily dose of NAC (250 mg/kg, p.o.) for 14 days and were injected with a single injection of Cp (7 mg/kg, i.p.) on Day 7. **Groups 4 (CGA group)**—rats received a daily dose of CGA (20 mg/kg, p.o.) for 14 days and were injected with a single injection of Cp (7 mg/kg, i.p.) on Day 7. **Group 5 (NAC + CGA group)**—rats received a daily dose of NAC (250 mg/kg, p.o.) and CGA (20 mg/kg, p.o.) for 14 days and were injected with a single injection of Cp (7 mg/kg, i.p.) on Day 7. Doses of Cp [79], NAC [50], and CGA [80] were chosen according to previous studies. The animals’ weights were recorded on the first and seventh days of the experiment to calculate the dose.

At the end of the experiment, rats were weighed then sacrificed after carbon dioxide-mediated anesthesia. Blood from the different groups was collected from the trunk, left to stand for 45 min, and then centrifuged at 1000× *g* for 15 min. Sera was stored in 2 mL Eppendorf tubes, divided into aliquots, and kept at −80 °C for biochemical analysis. Kidney tissues were dissected, washed with ice-cold phosphate-buffered saline, and weighted. Part of the kidney was homogenized using a tissue homogenizer (Omni International Inc., Kennesaw, GA, USA) and centrifuged at 10,000 rpm at 4 °C for 15 min. Then, the supernatants were stored at −80 °C until they were used to assess the oxidative stress, inflammatory, and apoptotic markers. Another part of the kidney was kept in liquid nitrogen for western blot analysis. Furthermore, histopathological examination was done on kidney samples obtained from the different treatment groups and treated immediately with 10% formaldehyde.

#### 4.2.2. Assessment of Nephrotoxicity Indices

The kidney index of the different treatment groups was calculated according to the following equation: (kidney weight/body weight) × 100 [81,82]. In addition, serum levels of creatinine [83] and blood urea nitrogen (BUN) [84] were assessed according to the previously described methods using the commercially available kits (United Diagnostics Industry, Dammam, Saudi Arabia). The manufacturer’s instructions were followed in the steps of the assay.

#### 4.2.3. Assessment of Histopathological Changes

For light microscopy, kidney tissues were kept in 10% formalin (pH 7.4) for 24 h before preparing specimens in molten paraffin blocks. Then, five micron-thick sections were cut and mounted using a rotary microtome. These sections were stained with hematoxylin and eosin (H and E) to detect kidney tubular damage. Histopathological samples were scored in a blinded manner from six non-overlapping microscopic fields (400× nearly 79,000 square micrometers, using Leica Biosystems) per a tissue section of a sample, then scored by an experienced histologist using the system adopted from El-Nabarawy et al., 2020 and Moon et al., 2022 as follows:

(Nil) for any sample with no abnormal cellularity, (+) if there were minor focal lesions seen in less than 50% of samples, (++) if more than 50% of samples showed mild lesions focally, (+++) for moderate diffused lesions in 75–100% of samples, and (++++) if total samples examined had severe diffused lesions [85,86].

#### 4.2.4. Assessment of Oxidative Stress Markers in Renal Tissues

The effect of Cp, NAC, and CGA on the oxidative stress markers in kidney tissues was evaluated. The renal level of total antioxidants was measured according to the previously described method by Ellman [87] using the Total Antioxidants Assay Kit (BioSource International Inc., Camarillo, CA, USA). In addition, lipid peroxidation was assessed using malondialdehyde assay kit (Biodiagnostic Co., Giza, Egypt) that measures the level of thiobarbituric acid-reactive substances (TBARS), and data were finally expressed as malondialdehyde equivalents [88]. Glutathione peroxidase (GPx) and Catalase enzyme activities were also measured in using a commercially available kit (Biodiagnostic Co., Giza, Egypt).

#### 4.2.5. Assessment of Renal Expression of Inflammatory Markers (NF-κB, TNF-α) Using ELISA

According to the manufacturer’s instructions, the expression of NF-κB and TNF-α in tissue homogenates of the different treatment groups was measured using specific assay ELISA Kit (BioSource International Inc., Camarillo, CA, USA).

#### 4.2.6. Assessment of Renal Expression of Apoptotic Markers (Bax/Bcl-2 Ratio) Using ELISA

The commercially available ELISA kits (BioSource International Inc., Camarillo, CA, USA) were used to measure the renal expressions of Bax and Bcl-2 and expressed as Bax/Bcl-2 ratio. The procedure was done according to the manufacturer’s instructions.

#### 4.2.7. Assessment of Protein Content

We measured protein contents in kidney homogenates according to the previously described method by Bradford [89] using bovine serum albumin as a standard.

#### 4.2.8. Western Blot Analysis of TLR4, NF-κB, NLRP3, Caspase-1, IL-1β, Caspase-3, and GSDMD Protein Expression in Kidney Tissues

Kidney specimens from each group were subjected to western blot analysis preparation and protein extraction. Then, protein concentrations were determined using the Direct Detect quantification assay technique. Protein extraction and quantification were fully described previously [90]. Separated protein gels were transferred to polyvinylidene difluoride (PVDF) membranes (0.2 μm, Immun-Blot^®^, Bio-Rad, Hercules, CA, USA). Membranes were incubated (for 24–48 h) at 4 °C in the primary antibodies diluted as described in tris buffer saline with tween (TBST buffer) with a ratio of 1:1000 for TLR4 (ab22048), NF-KB p65 (ab16502), NLPR-3 (ab263899), caspase-1 (ab179515), IL-1β (ab254360), Caspase-3 ”Asp175” (C.S9661), and GSDMD (ab219800), while anti-GAPDH (ab8245) was used as a housekeeping loading control antibody. The intensities of different protein bands were quantified densitometrically using the Image J software (NIH Image, Bethesda, MD, USA) and normalized against loading control (GAPDH).

### 4.3. Statistics

We used GraphPad Prism software version 5 (ISI^®^ software, San Diego, CA, USA) to analyze and present graphs. Data are represented as means ± SEM. One-way ANOVA was used to compare multiple groups statistically, followed by the Tukey–Kramer post-hoc test. The significance level was accepted at *p* < 0.05.

## 5. Conclusions

**Figure 7:**
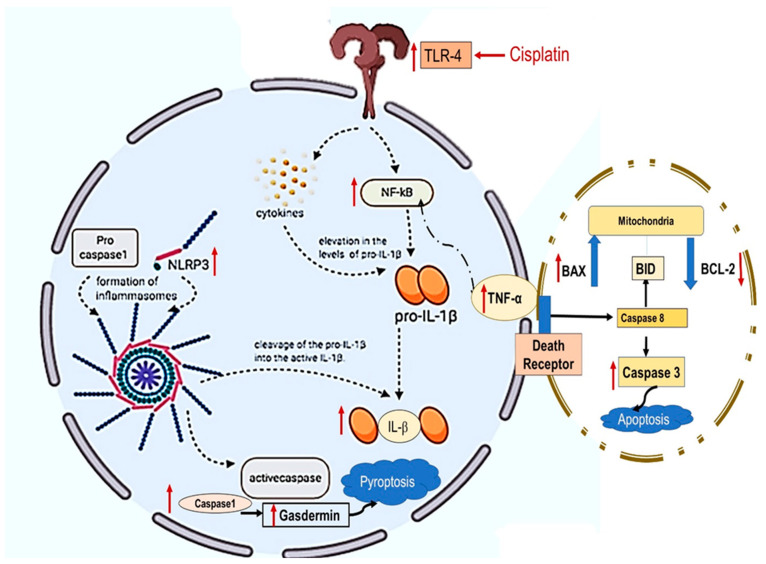
Cisplatin effect is represented as red arrows. TLR4: Toll-4 receptors; NF-κB: Nuclear factor-αB; NLPR3: Inflammasomes; IL-1β, and TNF-α; Tumor necrosis factor-α.

Collectively, the current study explored the promising nephroprotective effect of NAC and/or CGA against Cp-induced kidney damage and further elucidated the molecular mechanistic-signaling pathways implicated in their nephroprotection. Both NAC and CGA prevented the renal tubular massive insults through their antioxidant, anti-inflammatory, and antiapoptotic effects. Mechanistically, this present study was the first to suggest that the inhibition of TLR4/NLRP3 and caspase-1/GSDMD-signaling pathways might have been incorporated in both NAC and CGA nephroprotection. Consequently, chemotherapy-treated cancer patients may benefit from NAC and CGA as nephroprotective adjuvants (Figure 7).

## Figures and Tables

**Table 1:** Effect of NAC and/or CGA on Cisplatin-Induced Changes in Renal Function Indices and Oxidative Stress.

Treated Groups	Kidney Function Parameters			Oxidative Stress Markers			
	Kidney Index (%)	BUN (mg/dL)	Creatinine (mg/dL)	Lipid Peroxidation (nmol/mg Protein)	Total Antioxidant (mM)	Catalase (U/mg Protein)	Glutathione Peroxidase (mU/mL)
Control	0.96 ± 0.03	30.56 ± 3.250	0.31 ± 0.03	1.35 ± 0.18	2.18 ± 0.14	7.55 ± 0.61	2.52 ± 0.50
Cisplatin	1.36 ^a^ ± 0.16	238.90 ^a^ ± 29.09	2.89 ^a^ ± 0.45	2.72 ^a^ ± 0.24	1.24 ^a^ ± 0.07	1.45 ^a^ ± 0.57	37.00 ^a^ ± 6.15
NAC (250 mg/kg)	1.17 ± 0.11	62.76 ^b^ ± 6.45	1.26 ^b^ ± 0.32	1.29 ^b^ ± 0.06	2.11 ^b^ ± 0.29	3.60 ^a,b^ ± 0.57	11.60 ^b^ ± 1.96
CGA (20 mg/kg)	1.31 ± 0.14	82.31 ^b^ ± 11.07	1.49 ^b^ ± 0.20	1.69 ^b^ ± 0.14	1.70 ^b^ ± 0.12	2.43 ^a,b^ ± 0.09	23 ^a^ ± 3.48
NAC + CGA	1.12 ± 0.12	43.75 ^b^ ± 7.03	1.13 ^b^ ± 0.14	1.35 ^b^ ± 0.17	2.75 ^b^ ± 0.48	3.25 ^a,b^ ± 0.32	26 ^a^ ± 2.27
F, df, *p* value	6, 0.048 #	23.77, 4, 0.0001	9.5, 4, 0.0003	12.22, 4, 0.0001	5.23, 4, 0.0069	29.14, 4, 0.0001	18.20, 4, 0.001

Data are mean ± SEM (n = 5). ^a^ or ^b^: Statistically significant when compared to the control or cisplatin group, respectively, *p* < 0.05 using one-way ANOVA followed by Tukey–Kramer as post-hoc test. BUN: Blood Urea Nitrogen; NAC: N-acetylcysteine; CGA: Chlorogenic acid. # t = 2, two-tailed *p* value using student *t* test and all others column were not significant.

**Table 2:** Scoring of Histopathological Tissue Damage in Different Groups.

	Control	Cisplatin	Cisplatin + NAC	Cisplatin + CGA	Cisplatin + NAC + CGA
tubular degenerative changes	-	++++	++	+	-
tubular dilatation	-	+++	++	+	+
intraluminal casts	-	+++	-	-	-
inflammatory cells infiltrates	-	++	-	-	+

Histopathological samples were scored in a blinded manner from 6 non overlapping microscopic fields 400× (nearly 79,000 square micrometers, using Leica Biosystems), then scored by an experienced histologist as follows: (Nil) for any sample with no abnormal cellularity, (+) if there were minor focal lesions seen in less than 50% of samples, (++) if more than 50% samples showed mild lesions focally, (+++) for moderate diffused lesions in 75–100% of samples, and (++++) if total samples examined had severe diffused lesions.

## Data Availability

The authors confirm that the data supporting the findings of this study are available within the article.

## Abbreviations

AKI: acute kidney injury; BUN: blood urea nitrogen; CGA: chlorogenic acid; Cp: Cisplatin; Damps: endogenous-associated molecular patterns; GSDMD: gasdermin D; NAC: N-acetylcysteine; NF-kB: nuclear factor-kB; NLRP3: NLR family pyrin domain-containing 3; Pamps: pathogen-associated molecular pattern; Pro-IL-1β: pro-interleukin (IL)-1beta; PRR: pattern recognition receptors; ROS: reactive oxygen species; TBARS: thiobarbituric acid reactive substances; TLR4: Toll-4 receptors.
